# Rumen–Plasma–Milk Metabolomics Profiling Revealed Metabolic Alterations Associated with Milk Fat Synthesis in Chinese Holstein Cows

**DOI:** 10.3390/ani16081136

**Published:** 2026-04-08

**Authors:** Huimin Zhang, Sam Carie Kollie, Tianyu Xia, Zhendong Yang, Marazi Tanaka Ian, Ahmed A. Elolimy, Wanqiong Wang, Dongsheng Lu, Yi Li, Mingxun Li, Juan J. Loor, Yongjiang Mao, Zhangping Yang

**Affiliations:** 1 Key Laboratory for Animal Genetics, Breeding, Reproduction and Molecular Design of Jiangsu Province, College of Animal Science and Technology, Yangzhou University, Yangzhou 225009, China; minmin-911@163.com (H.Z.);; 2 Technology Innovation Center of Meat and Meat Products, State Administration for Market Regulation, Jinan 250101, China; 3 Department of Integrative Agriculture, College of Agriculture and Veterinary Medicine, United Arab Emirates University, Al Ain P.O. Box 15551, United Arab Emirates; 4 Department of Animal Sciences & Division of Nutritional Sciences, University of Illinois, Urbana, IL 61801, USA

**Keywords:** milk fat synthesis, metabolomics, rumen fluid, plasma, milk, malic acid, bovine mammary epithelial cells

## Abstract

Milk fat synthesis in dairy cows is a complex process affected by ruminal fermentation, systemic metabolism, and mammary gland activity. To explore the metabolic interplay across these systems, a multi-tissue metabolomics approach (rumen fluid, plasma, and milk) using ultra-high-performance liquid chromatography–mass spectrometry was used to identify metabolic differences between Chinese Holstein cows with high (H-MF, 5.82 ± 0.41%) and low (L-MF, 3.60 ± 0.12%) milk fat content under the same diet. The bovine mammary epithelial cells (BMECs) were also cultured to evaluate the impact of a key metabolite, malic acid (MA), on lipid metabolism. Our findings reveal distinct metabolic profiles across rumen fluid, plasma, and milk, with 96, 109, and 79 differential metabolites, respectively, between the L-MF and H-MF groups. In rumen fluid, H-MF cows showed higher levels of lauric acid and succinic acid, linked to fatty acid biosynthesis, while the L-MF cows had elevated citraconic and orotic acids, associated with amino acid metabolism and liver stress. Plasma from the H-MF cows contained higher *β*-hydroxybutyric acid, methionine sulfoxide, and phosphatidylcholine, supporting lipogenesis, whereas L-MF plasma showed increased 3-hydroxy-L-proline, indicating tissue catabolism. In milk, the L-MF cows had higher MA, while the H-MF cows exhibited elevated L-carnitine, linked to fatty acid β-oxidation. Metabolite trend analysis during rumen fluid–plasma–milk showed that 211 metabolites were classified into 8 profiles. Profile 1 had the largest number of metabolites whose levels were down-regulated from rumen to plasma and enriched in lipid metabolism. Profile 3 (mainly related to amino acid metabolism) and profile 4 (mainly related to energy metabolism) exhibited opposite trends from plasma to milk. In vitro, 200 μM of MA reduced the triglyceride content in BMECs and down-regulated lipogenic genes and their protein expression levels (fatty acid synthase, stearoyl-CoA desaturase and sterol regulatory element binding protein 1). These results highlight how rumen fluid, plasma, and milk metabolites collectively influence milk fat synthesis, with MA acting as a key regulator of lipid metabolism in mammary epithelial cells.

## Simple Summary

This study used untargeted metabolomics of rumen fluid, plasma and milk to explore the metabolic alterations associated with milk fat synthesis in Chinese Holstein cows. Results show distinct metabolic profiles across the rumen fluid, plasma, and milk between the cows with high and low milk fat content under the same diet. Organic acids, lipids and amino acids were the main differential metabolites. Metabolite trend analysis during rumen fluid–plasma–milk showed that 211 metabolites were classified into 8 profiles. Profile 1 showed lipid metabolites down-regulated from rumen fluid to plasma. Trends of amino acids and energy metabolisms were reversed from plasma to milk. In vitro, a key metabolite (malic acid) reduced the triglyceride content in bovine mammary epithelial cells and down-regulated the expression levels of lipogenic genes/proteins. This study demonstrates that multi-tissue metabolites collectively regulate milk fat synthesis, with malic acid as a key lipid metabolism regulator in dairy cows, offering potential insights into optimizing dairy cow nutrition and milk quality.

## 1. Introduction

Milk fat synthesis is a biological process influenced by a combination of dietary intake, genetic predisposition, and physiological changes associated with lactation stages [1]. Understanding and optimizing this process is crucial for improving milk quality and productivity, and this is also linked to the economic interests of dairy farms. Milk fat consists predominantly of triglyceride (TAG) containing more than 400 individual fatty acids (FAs) [2]. Milk FAs originate from two general sources: de novo FA synthesis in the mammary gland and transfer of FA from the blood [3]. The FAs from C4 to C14 and half of C16 are de novo synthesized from short-chain FAs (SCFAs), such as acetate and butyrate produced by the ruminal microbiome, whereas the other half of C16 and those higher in milk are derived either from diet or transfer of FAs from TAG in the blood [4]. In dairy cows, the glycerol backbone of TAG emerges from an elegant metabolic process: glucose, forged from rumen-derived propionate through hepatic gluconeogenesis, transforms into glycerol-3-phosphate within the mammary gland, uniting with FAs to craft the triacylglycerols that define milk fat [5]. Key enzymes, such as acetyl-CoA carboxylase (ACC) and fatty acid synthase (FASN), facilitate the synthesis of FAs in the mammary gland using acetate and, to a lesser extent, butyrate, produced in the rumen [6].

Acetate supplementation in the diet of dairy cows increased plasma *β*-hydroxybutyric acid (BHB) concentration and increased milk fat yield, especially through de novo FA synthesis [5]. Several studies have revealed that the metabolisms in rumen and plasma are closely related to milk fat metabolism, but an interplay among these metabolic processes remains to be more explored at the whole-body level [7,8].

Metabolomics has facilitated the profiling of metabolic alterations and the identification of essential metabolic pathways associated with milk fat production [9]. The ruminal microbiome and their fermentation products are crucial for digesting feed substrates and producing volatile fatty acids (VFAs), which serve as essential energy sources and precursors for milk FA synthesis in dairy cows [10]. The blood metabolome captures systemic metabolic dynamics and nutrient delivery to the mammary gland, while the milk metabolome reflects the metabolic products synthesized and secreted by the mammary gland during lactation in dairy cows [11].

The multi-tissue approach has been shown to uncover complex metabolic interactions and regulatory mechanisms, as demonstrated in many studies [12,13]. A metabolomics comparison of rumen fluid and milk in dairy cows showed that organic acid and carbohydrate metabolites exhibited the highest concentrations in rumen fluid and milk, respectively, and some metabolites may be useful for predicting metabolic diseases and milk quality [14]. Plasma and milk metabolomics of dairy cows indicated that heat stress triggered more prominent changes in the milk than in the plasma metabolome, with consistent results in milk suggesting increased muscle catabolism, as reflected by increased creatinine, alanine and citrulline levels [12].

Based on previous studies, it was hypothesized that the multi-tissue metabolomics approach can reveal how ruminal fermentation products are utilized in plasma and then subsequently utilized in milk for fat synthesis. Further, no studies have combined the metabolome of rumen fluid, plasma and milk to reveal the process of milk fat synthesis. To test our hypothesis, we employed a multi-tissue metabolomics (rumen fluid, plasma, and milk) approach to investigate the metabolic mechanisms underlying milk fat synthesis in Chinese Holstein cows. After a comparative analysis of the differential metabolites in single tissue (rumen fluid, plasma, and milk) and rumen fluid–plasma–milk trend analyses, the common metabolite (malic acid, MA) was selected, and its effect on TAG synthesis was explicated with bovine mammary epithelial cells (BMECs).

## 2. Materials and Methods

### 2.1. Experimental Animals and Sample Collection

The experimental procedures of dairy cows were approved by the Institutional Animal Care and Use Committee (IACUC) of the College of Animal Science and Technology, Yangzhou University. Dairy herd improvement (DHI) data (from August 2020 to January 2021) of 532 lactating Chinese Holstein cows in the Wanhe dairy farm in Jiangsu Province of China were collected and analyzed. The cows in our study were housed in free stalls and provided with the same total mixed ration (TMR) (Table S1). This diet was formulated in accordance with NRC (2001) guidelines to meet or exceed the nutrient specifications for lactating dairy cows [15]. All lactating dairy cows in this dairy farm have used this feed formula from 1 November 2020. Five cows with high average milk fat content (5.08 ± 0.23%, H-MF group) and five with low average milk fat content (3.71 ± 0.13%, L-MF group) based on the DHI data from August 2020 to January 2021 were selected in our previous study [16]. As shown in Figure S1 and Table S2, from August 2020 to January 2021, the mean milk fat content of the L-MF group in autumn and winter was lower than the H-MF group (*p* < 0.01). Cook’s distance was calculated for each cow (Figure S2). Both values in autumn and winter were below the reference threshold (4/*n* = 0.4), showing no outlier in the selected cows. All cows were in similar parity (L-MF: 1.80 ± 0.37, H-MF: 1.80 ± 0.37), similar daily milk yield (L-MF: 28.54 ± 1.90 kg, H-MF: 27.96 ± 5.34 kg), and similar stage of lactation (L-MF: 257.80 ± 55.90 d, H-MF: 218.80 ± 77.98 d). The determination of daily dry matter intake (DMI), diet composition and nutrient levels were also reported as previously described [16]. Approximately two hours after morning feeding on 16 January 2021, milk, rumen fluid, and blood samples were collected. Milk samples in the morning, midday, and evening were collected and mixed. Each sample was separated into two sterile tubes. One was immediately placed in liquid nitrogen and stored at −80 °C for metabolomics analysis. The other one was stored at 4 °C for composition analysis. The sampling process and milk composition analysis were conducted as described by our previous study [16].

### 2.2. Plasma Extraction and Biochemical Index Analysis

Blood was harvested from the tail-head region. Plasma was obtained by centrifugation of EDTA anticoagulant-coated tubes at 2000× *g* for 20 min. Concentrations of glucose, TAG, total cholesterol, high-density lipoprotein cholesterol, low-density lipoprotein, and BHB and non-esterified fatty acids (NEFAs) were measured by an Automatic Biochemistry Analyzer (HITACHI 7180, Tokyo, Japan).

### 2.3. Metabolomic Analysis

Rumen fluid, plasma and milk samples were thawed at room temperature. A total of 100 μL samples were mixed with 500 μL of prechilled 80% methanol and 0.1% formic acid by vortex for 30 s. Then, the solution was incubated on ice for 5 min and centrifuged at 15,000× *g* for 20 min at 4 °C. The supernatant was diluted to a final concentration containing 53% methanol by water with liquid chromatography–mass spectrometry grade. After centrifugation at 15,000× *g* for 20 min at 4 °C, the supernatant was transferred to injection vials for subsequent ultra-high-performance liquid chromatography–mass spectrometry (UHPLC-MS/MS) analysis.

UHPLC-MS/MS analyses were conducted using a Vanquish UHPLC system (Thermo Fisher, Bremen, Germany) coupled with an Orbitrap Q ExactiveTMHF-X mass spectrometer (Thermo Fisher, Bremen, Germany). UHPLC was performed with a Hypesil Gold column (100 × 2.1 mm, 1.9 μm, Thermo Fisher, Waltham, MA, USA), using eluent A and eluent B as the mobile phase at a flow rate of 0.2 mL/min (Table 1). Eluents A and B were the same as a previous study [16]. The column temperature was maintained at 40 °C, and the sample injection volume was 3.0 μL. The mass spectrometer was operated in POS/NEG mode, with a spray voltage of 3.2 kV, a capillary temperature of 320 °C, a sheath gas flow rate of 40 arb and an aux gas flow rate of 10 arb.

### 2.4. Bioinformatics Analysis

The processed data were assessed by random forest analysis using the R v4.4.0 package randomForest 4.6-14 [17]. Random forest was used to build the prediction model to identify the potential diagnostic biomarkers. Metabolites were ranked according to their importance in separating the milk fat groups based on a mean decrease accuracy (MDA) score in random forest results. Student’s *t*-test was used to analyze the differences in metabolites. The metabolites with MDA > 0.004 and *p* < 0.05 were considered as the differential metabolites. The Receiver Operating Characteristic (ROC) curve was analyzed using the R package pROC 1.18.0, according to the significant different metabolites between the two groups [18]. The area under the curve (AUC) was computed via numerical integration of the ROC curves. The closer the AUC value is to 1, the higher the accuracy test is [19]. The Short Time-Series Expression Miner (STEM, version 1.2.2b) software generated the trend profiles [20]. Metabolites were mapped to KEGG metabolic pathways for annotation and enrichment analysis by MetaboAnalyst 5.0 [21].

### 2.5. Cell Culture and Treatment

The BMECs were isolated, purified, and cultured from lactating Chinese Holstein cows, then preserved in our laboratory. Cell isolation and culture of BMECs was conducted as previously described [22]. The BMECs were incubated in the presence of various concentrations (0, 50, 100, 200, and 400 μM) of DL-MA (Sigma, St. Louis, MO, USA) for 24 h to investigate the dose effect of MA. The cells were incubated at 37 °C in 5% CO_2_. BMECs without MA treatment served as a negative control (NC). Each concentration group consisted of 3 replicates.

### 2.6. Cell Viability and Proliferation Determination

After BMECs were treated with different concentrations of MA for 24 h, cell viability was measured using a cell counting kit-8 (CCK-8; Vazyme, Nanjing, China), and cell viability was measured using EdU cell proliferation assays (Beyotime Biotechnology Inc., Shanghai, China). The specific experimental procedures were the same as a previous study [16]. Each concentration group consisted of 3 replicates.

### 2.7. Intracellular TAG Content

After incubation with 200 μM of MA for 24 h, BMECs were rinsed twice with PBS and used for lipid analysis. Intracellular TAG was analyzed with a tissue triglyceride assay kit (Applygen, Beijing, China) according to the manufacturer’s instructions. TAG content was quantified by measuring absorbance at 550 nm with a Multiskan SkyHigh microplate reader (Thermo Fisher, Woodlands, Singapore) and normalized to protein concentration, determined using the Pierce^®^ BCA protein assay kit (Thermo Scientific, Rockford, IL, USA).

### 2.8. RT-qPCR and Western Blotting

The procedure of RT-qPCR was conducted as previously described [22]. Primer sequences are described in Table S3. *β*-actin was used as the control gene to normalize the amount of cDNA in the qPCR reaction. The data were analyzed using the relative quantification (2^−ΔΔCt^) method [23].

Protein extraction and quantification were carried out following a previously established method [22]. Subsequently, proteins were separated by SDS-PAGE, electrotransferred onto PVDF membranes (Millipore, Bedford, MA, USA), and blocked with skim milk. The membranes were then incubated with primary antibodies targeting FASN (Proteintech, Rosemont, IL, USA, 10624-2-AP, 1:1000), SREBF1 (Proteintech, Rosemont, IL, USA, 14088-1-AP, 1:1000), PPAR Gamma (Proteintech, Rosemont, IL, USA, 16643-1-AP, 1:1000), and β-actin (Beyotime Biotechnology Inc., Shanghai, China, AF5003, 1:1000), followed by incubation with an HRP-conjugated goat anti-rabbit IgG secondary antibody (Beyotime Biotechnology Inc., Shanghai, China, A0208, 1:1000). Protein signals were visualized using the Pierce ECL Western blotting substrate (Thermo Fisher, Rockford, IL, USA) and captured with a ChemiDoc XRS+ digital imaging system (Bio-Rad, Hercules, CA, USA).

### 2.9. Statistical Analysis

The differences between the L-MF and H-MF groups were compared with Student’s *t*-test in the SPSS 27.0 software [24]. Cook’s distance analysis was conducted using regression analysis in the SPSS 27.0 software. The data are shown as the means ± standard errors (SEs). Differences were considered statistically significant when *p* < 0.05.

## 3. Results

### 3.1. Milk Composition

Data describing the milk composition of the experimental cows have been reported in our previous study [16]. Briefly, the milk fat content of the H-MF group on the sampling day was 5.82 ± 0.41%, which was greater than the L-MF group (3.60 ± 0.12%) (*p* < 0.01). No significant differences were observed in milk protein, lactose, solids-not-fat content, MUN, and SCS between the two groups on the sampling day (*p* > 0.05). And there was no significant difference in DMI intake (L-MF: 22.50 ± 1.85 kg/d, H-MF: 21.57 ± 1.80 kg/d, *p* > 0.05).

### 3.2. Plasma Biochemical Index Analysis

Most of the plasma biochemical index (glucose, TAG, total cholesterol, high-density lipoprotein cholesterol, low-density lipoprotein cholesterol and NEFA) did not differ (*p* > 0.05) between the two groups (Table 2). A higher concentration of BHB was observed in the plasma of H-MF compared with the L-MF group (*p* < 0.05).

### 3.3. Rumen Fluid Metabolite Analysis

**Figure 1:**
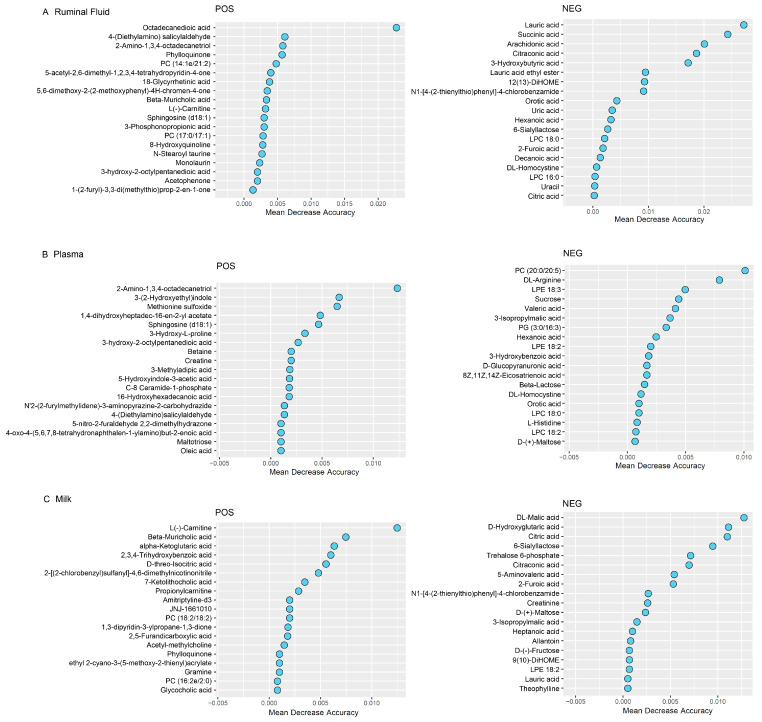
Random forest analysis identified a unique metabolomic signature between L-MF and H-MF groups. A higher mean-decrease-accuracy (MDA) value indicates a greater predictive value. (**A**) rumen fluid; (**B**) plasma; (**C**) milk.

**Figure 2:**
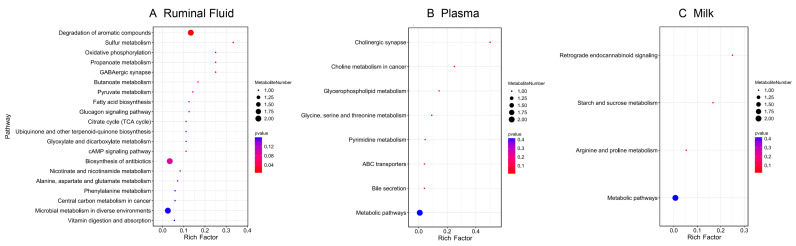
KEGG analysis of differential metabolites. (**A**) Rumen fluid; (**B**) plasma; (**C**) milk.

A total of 1577 metabolites in POS mode and 990 in NEG mode were identified among the rumen fluid–plasma–milk samples. In rumen fluid, 96 metabolites differed significantly between the L-MF and H-MF groups based on the *t*-test (fold change ≠ 1 and *p* < 0.05) (Table S4). Random forest analysis identified a unique metabolic signature between the L-MF and H-MF groups, with a predictive accuracy of 40% (POS mode) and 80% (NEG mode). AUC values from ROC in POS and NEG modes were 0.40 and 0.96, indicating that the NEG mode yielded a more accurate prediction (Table 3). Therefore, 9 significant metabolites were defined as the potential key metabolites (MDA > 0.004 and *p* < 0.05) in rumen fluid (Figure 1A, Table 4). Compared with the L-MF group, the levels of octadecanedioic acid, lauric acid, succinic acid, lauric acid ethyl ester and 12(13)-DiHOME were greater in the H-MF group, while citraconic acid, 3-Hydroxybutyric acid, N1-[4-(2-thienylthio)phenyl]-4-chlorobenzamide and orotic acid were lower. The KEGG pathway analysis revealed that these metabolites were enriched in propanoate metabolism, butanoate metabolism, pyruvate metabolism, fatty acid biosynthesis and citrate cycle (Figure 2A).

### 3.4. Plasma Metabolite Analysis

In plasma, 109 metabolites differed significantly between the L-MF and H-MF groups (fold change ≠ 1, *p* < 0.05) (Table S5). In the random forest analysis, the prediction accuracy of POS and NEG modes was 70.00% and 60.00%, respectively, and the ROC curve analysis showed that the AUC values were 0.60 and 0.84, respectively, which indicated that both modes’ prediction accuracy was high and could effectively distinguish the L-MF and H-MF groups (Table 3). After screening with MDA > 0.004 and *p* < 0.05 (Figure 1B, Table 4), 7 differential metabolites were identified. Compared with the L-MF group, the up-regulated differential metabolites in the H-MF group were methionine sulfoxide, PC (20:0/20:5), DL-arginine and LPE 18:3, while 2-Amino-1,3,4-octadecanetriol, 3-(2-Hydroxyethyl) indole, and 3-Hydroxy-L-proline were down-regulated. These metabolites were enriched in the cholinergic synapse, glycerophospholipid metabolism and glycine, serine and threonine metabolism (Figure 2B).

### 3.5. Milk Metabolite Analysis

In milk, 79 metabolites differed significantly between the L-MF and H-MF groups (fold change ≠ 1, *p* < 0.05) (Table S6). Random forest classification prediction accuracy was 70.00% and 60.00% in the POS and NEG modes, respectively. The AUC value of both modes reaches 0.76, indicating the high accurate prediction of both modes (Table 3). Compared with the L-MF group, the differential metabolites up-regulated in the H-MF group were L(-)-Carnitine, beta-muricholic acid and trehalose 6-phosphate, while MA was down-regulated (Figure 1C, Table 4). These metabolites were mainly enriched in retrograde endocannabinoid signaling, starch and sucrose metabolism, and arginine and proline metabolism (Figure 2C).

### 3.6. Trend Analysis of Rumen Fluid–Plasma–Milk Metabolites

**Figure 3:**
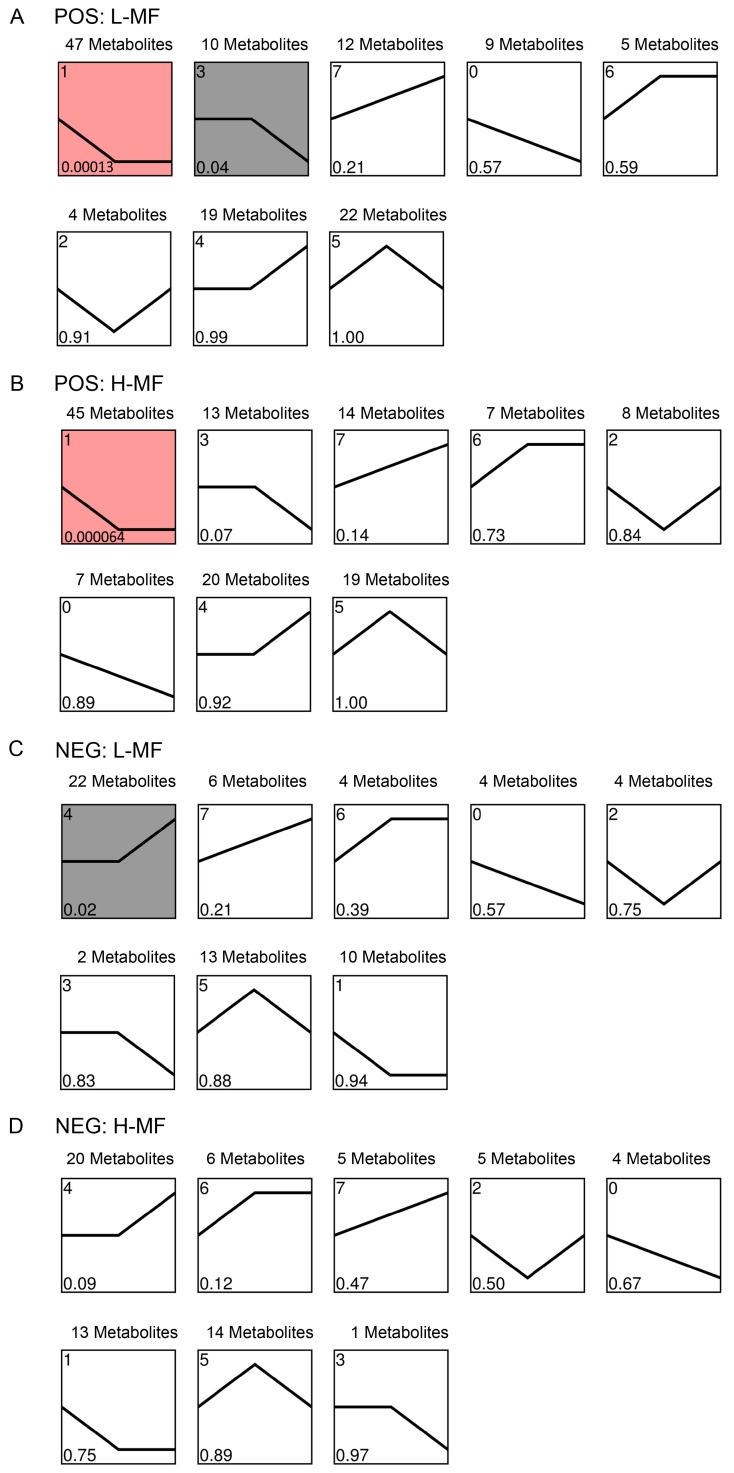
Trend analysis of the variation pattern of metabolites during rumen fluid–plasma–milk from dairy cows with different milk fat contents. The bend line shows the normalized level of metabolite in each profile. The number of metabolites belonging to each pattern is indicated above the box. The profile number is indicated in the upper-left corner of the box, and the *p* value is indicated in the lower-left corner of the box. Profiles with red and grey backgrounds indicate that the variation pattern of metabolites was significantly enriched in this pattern, with *p* < 0.01 and *p* < 0.05, respectively. (**A**) Metabolites in POS from L-MF group; (**B**) metabolites in POS from H-MF group; (**C**) metabolites in NEG from L-MF group; (**D**) metabolites in NEG from H-MF group.

A trend analysis of the metabolites was performed using the STEM software 1.0 to further compare the variation pattern of metabolites during rumen fluid–plasma–milk from dairy cows with different milk fat contents. In the POS mode, a Venn diagram displayed 8 unique metabolites in the trend analysis of the L-MF group and 12 unique metabolites in the H-MF group, and both groups shared 121 metabolites (Figure S3, Table S7). In the NEG mode, 66 metabolites were enriched in the trend analysis of the L-MF group, 69 metabolites were enriched in the H-MF group, and 65 metabolites were shared by both groups (Figure S3, Table S8). Metabolites were categorized into 8 profiles (Figure 3), including three up-regulated patterns (profiles 4, 6 and 7), three down-regulated patterns (profiles 0, 1 and 3) and two mixed patterns (profiles 2 and 5). In the POS mode (Figure 3A,B), 47 and 10 metabolites were significantly enriched in profiles 1 and 3 of the L-MF group (*p* < 0.05); in contrast, 45 metabolites were significantly enriched in profile 1 of the H-MF group (*p* < 0.05). In the NEG mode (Figure 3C,D), profile 4 had the largest numbers of metabolites. A total of 22 metabolites were significantly enriched in profile 4 of the L-MF group (*p* < 0.05), while the H-MF group had no significantly enriched profile.

**Figure 4:**
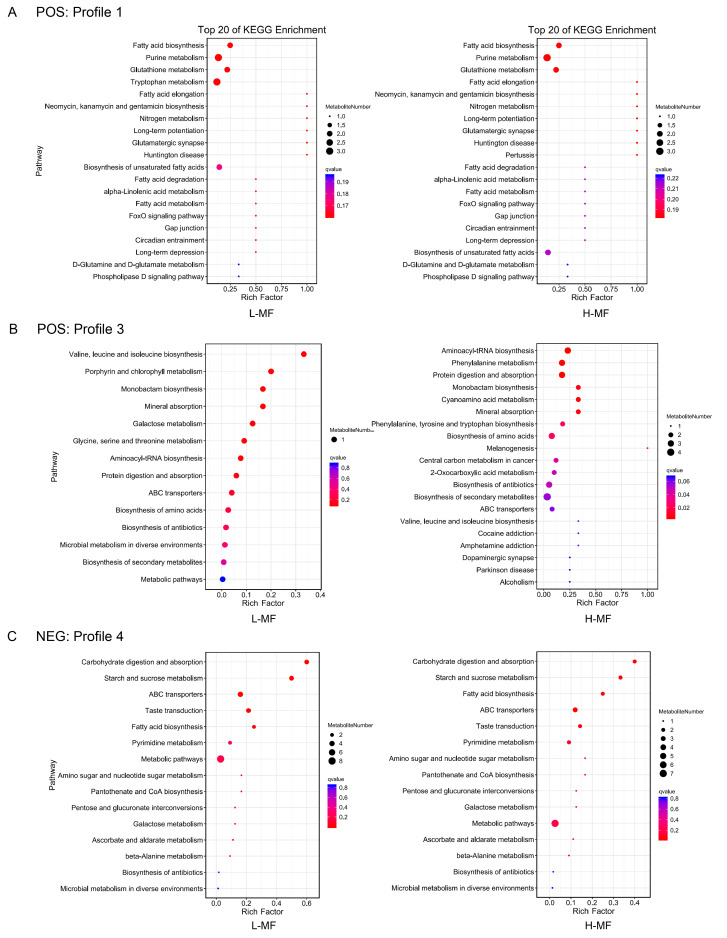
KEGG enrichment analysis of metabolites in the trend analysis. (**A**) Profile 1 in POS mode; (**B**) profile 3 in POS mode; (**C**) profile 4 in NEG mode.

In the POS mode (Figure 3A,B), profile 1 had the largest numbers of metabolites whose levels were down-regulated from rumen to plasma, while their levels were similar between plasma and milk samples. Most metabolites in profile 1 were annotated as fatty acyls, indoles and derivatives, and imidazopyrimidines and enriched in fatty acid biosynthesis, purine metabolism, glutathione metabolism and fatty acid elongation (*p* < 0.05) (Figure 4A). The specific and significant enrichment pathway in the L-MF group was tryptophan metabolism, and the metabolites kynuuric acid, 5-hydroxyindole-3-acetic acid and 3-(2-hydroxyethyl) indole were enriched in this pathway.

In the POS mode (Figure 3A,B), the metabolites included in profile 3 showed similar levels between rumen and plasma samples, and then down-regulated in milk. Most metabolites in profile 3 were classified as indoles and derivatives, carboxylic acids and derivatives, and organooxygen compounds, and they participated in KEGG pathways, such as valine, leucine and isoleucine biosynthesis, monobactam biosynthesis, and mineral absorption (*p* < 0.05) (Figure 4B).

In the NEG mode (Figure 3C,D), profile 4 had the largest number of metabolites whose levels were up-regulated from plasma to milk, indicating that the changes in these metabolites occurred from plasma to milk. Most metabolites in profile 4 were annotated as fatty acyls, organooxygen compounds and diazines, which were enriched in carbohydrate digestion and absorption, starch and sucrose metabolism, fatty acid biosynthesis and ABC transporters (*p* < 0.05) (Figure 4C).

In profile 1 of the POS mode, 7 and 5 metabolites were found specifically in the L-MF and H-MF groups, respectively (Table 5). Among them, 9-oxo-octadecanoic acid was enriched in linoleic acid metabolism (H-MF group), and kynurenic acid participated in tryptophan metabolism, which was significantly enriched in the L-MF group (*p* < 0.05). In profile 3 of the POS mode, LPC 18:3 was found specifically in the L-MF group, and 2-Hydroxycinnamic acid, L-Tyrosine, L-Phenylalanine and D-(+)-Proline were found specifically in the H-MF group, which were enriched in the phenylalanine metabolism. In profile 4 of the NEG mode, DL-Malic acid (DL-MA), methylsuccinic acid and D-(+)-maltose were found specifically in the L-MF group, and ethyl-D-glucuronide was found specifically in the H-MF group.

### 3.7. Effect of MA on Cell Viability and Proliferation of BMECs

**Figure 5:**
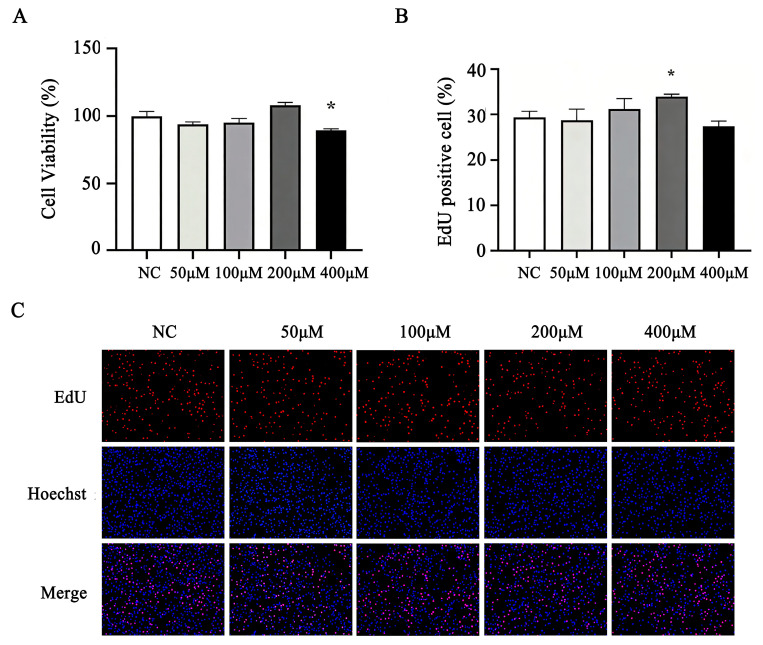
Effect of different concentrations of MA on the cell viability and proliferation of BMECs. (**A**) CCK-8 analysis; (**B**,**C**) EdU incorporation assay. * *p* < 0.05.

As reported in Figure 5A, 400 μM of MA significantly inhibited BMECs’ viability (*p* < 0.05), while no difference was observed between NC and lower doses of MA-treated cells (50, 100 and 200 μM). The EdU incorporation assay revealed that the percentage of EdU positive BMECs was increased by treatment with 200 μM of MA for 24 h (*p* < 0.05), while no difference was observed between NC and other doses of MA-treated BMECs (Figure 5B,C).

### 3.8. Effect of MA on Lipid Metabolism in BMECs

**Figure 6:**
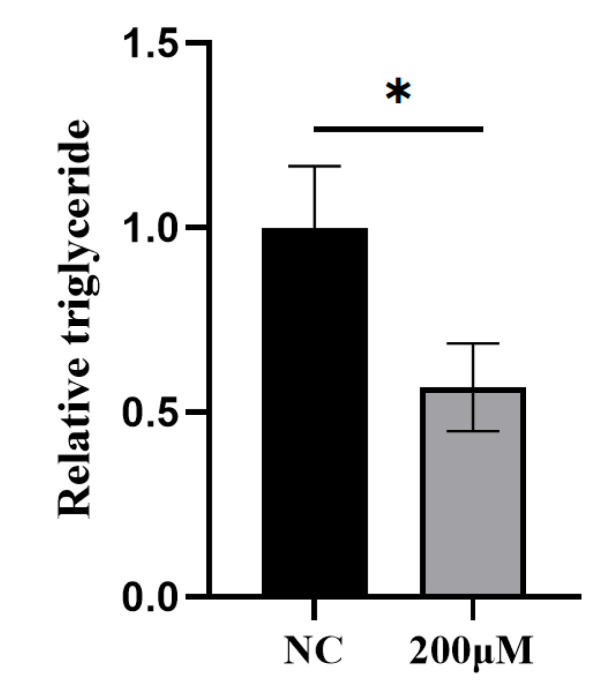
Analysis of TAG content in BMECs. * *p* < 0.05.

As reported in Figure 6, treatment with 200 μM of MA for 24 h dramatically decreased the TAG content in BMECs (*p* < 0.05).

**Figure 7:**
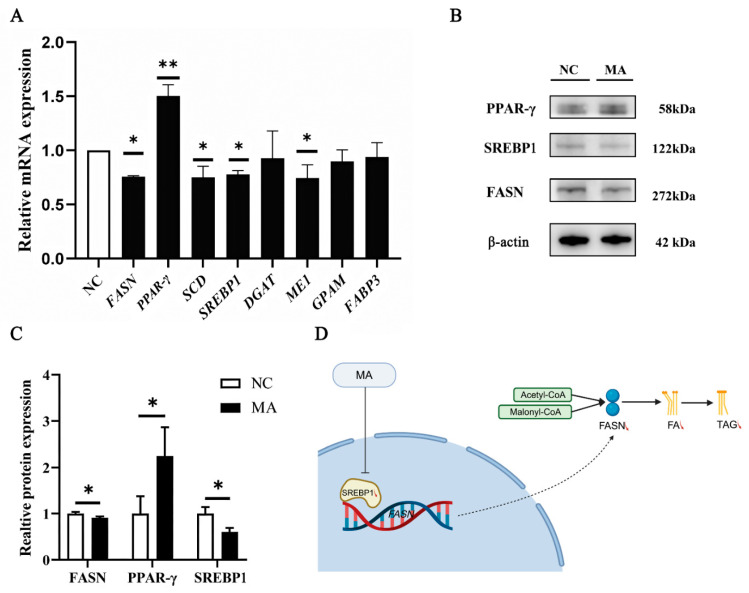
Expression of genes and proteins related to lipid metabolism in BMECs treated with 200 μM of MA. (**A**) The relative mRNA expression level of genes related to lipid metabolism after 200 μM of MA treatment in BMECs; (**B**,**C**) Western blotting analysis of the level of proteins related to lipid metabolism after 200 μM of MA treatment in BMECs. (**D**) Graphical representation of the effect of MA on TAG synthesis in BMECs, the red arrow indicated down-regulated. This image was created with bioRender.com (accessed on 12 November 2025). * *p* < 0.05; ** *p* < 0.01.

Compared with the NC group (Figure 7A), treatment with 200 μM of MA in BMECs dramatically down-regulated the mRNA abundance of *FASN*, *stearoyl-CoA desaturase* (*SCD*), *sterol regulatory element binding protein 1* (*SREBP1*) and *malic enzyme* 1 (*ME1*) (*p* < 0.05). However, the mRNA abundance of peroxisome proliferators-activated receptor gamma (*PPARγ*) was increased by 200 μM of MA treatment (*p* < 0.05). There was no significant change in the abundance of *diacylglycerol acyltransferase* 1 (*DGAT1*), *glycerol-3-phosphate acyltransferase* (*GPAM*) and *fatty acid-binding protein 3* (*FABP3*) (*p* > 0.05).

As reported in Figure 7B,C, the protein expression levels of FASN and SREBP1 were significantly decreased, whereas the protein expression level of PPARγ was significantly increased by 200 μM of MA treatment.

## 4. Discussion

### 4.1. Effect of Rumen Fluid Metabolite on Milk Fat Content

Metabolomics studies have shown that shifts in ruminal metabolites can significantly impact milk composition, with certain compounds linked to reduced milk fat synthesis [9]. In our study, we found that some ruminal metabolites, such as citraconic acid, orotic acid, 3-Hydroxybutyric acid, and N1-[4-(2-thienylthio)phenyl]-4-chlorobenzamide, were significantly higher in the L-MF cows. Citraconic acid and orotic acid have been identified as markers of altered amino acid metabolism and liver stress in dairy cows, both of which can negatively influence milk fat production. Specifically, citraconic acid is known to interact with metabolic pathways involving amino acid catabolism, potentially leading to imbalances that detract from efficient fat synthesis in mammary cells [11]. Similarly, orotic acid is an intermediary in pyrimidine synthesis, and its elevated level is often associated with hepatic stress and disrupted lipid metabolism, which are detrimental to milk fat content [25,26].

3-Hydroxybutyric acid, a ketone body, is indicative of a cow’s mobilization of body fat reserves, especially under energy-deficient conditions, such as early lactation. High levels of 3-hydroxybutyric acid signal metabolic stress and are often linked to reduced milk fat synthesis, as the mammary gland may prioritize energy preservation over milk fat production in response to elevated ketones [27]. Indeed, Lei et al. found that elevated ketone bodies in dairy cows correlated with a decrease in milk fat content due to metabolic adjustments [28].

Under the same diet in this study, concentrations of some FAs in rumen were elevated in the H-MF group compared with the L-MF group. These compounds, such as lauric acid, octadecanedioic acid, and lauric acid ethyl ester, could be transported by the portal venous system and might contribute to milk fat synthesis [16].

As for the function of key metabolites in the rumen, MA is a four-carbon dicarboxylic acid and is an intermediate in the succinate–propionate pathway of ruminal bacteria [29]. Many studies have found that MA supplementation in the feed can increase the concentration of VFAs in the rumen of dairy cows without affecting milk composition [30,31]. MA supplementation in lactating cow diets was also effective at increasing microbial N production and microbial efficiency measured in vitro and milk yield [29]. But no difference was observed in the ruminal VFA concentration (acetate, propionate, etc.) between the L-MF and H-MF groups [16].

### 4.2. Effect of Plasma Metabolite on Milk Fat Content

In ruminants, acetate and butyrate are produced through ruminal microbial carbon degradation. Butyrate is converted to BHB in the rumen wall and enters the circulatory system. The mammary gland uses acetate and BHB in the blood for the de novo synthesis of FAs in milk fat [32]. Sakowski et al. reported that BHB in the blood was positively correlated with milk fat content in dairy cows (r = 0.34) [33]. Moreover, an in vitro experiment has confirmed that the addition of BHB to BMECs increased the relative mRNA abundance of lipogenic genes (*FASN* and *SREBP1*) and cytosolic TAG content [34,35]. These reports agree with the higher BHB concentration detected in the plasma of the H-MF group, indicating that BHB in blood could be transported to the mammary gland to promote milk fat synthesis. When the plasma concentration of BHB exceeds 1 200 μmol/L, the dairy cow was assumed to be hyperketonemia [36]. But the plasma concentrations of the BHB of the cows used in our study was much lower than this threshold, so no ketosis was observed during our experiment.

In this study, plasma metabolomics have identified several metabolites. The levels of methionine sulfoxide, PC (20:0/20:5), DL-arginine, and LPE 18:3 were significantly higher in the H-MF cows. Amino acids play critical roles in lipid metabolism. For example, methionine and arginine are essential in processes supporting lipogenesis. Methionine provides methyl groups necessary for phospholipid and TAG synthesis, both important components of milk fat [37]. Arginine, meanwhile, contributes to lipid synthesis by promoting blood flow, ensuring that lipid substrates reach mammary tissue efficiently [38]. These amino acids act not only as building blocks for protein synthesis but also support fat production by enhancing the nutrient and precursor supply needed for milk fat synthesis.

Phosphatidylcholine (PC), a type of phospholipid, plays a direct role in lipid transport and cellular membrane formation, crucial for mammary gland lipid synthesis [39]. This particular PC species, PC (20:0/20:5), may serve as an FA transporter, facilitating the efficient transfer of FAs from plasma to the mammary gland for milk fat production. Lysophosphatidylethanolamine (LPE) 18:3, a bioactive lipid, influences lipid synthesis by modulating enzymes involved in FA and TAG formation [40]. LPE can influence lipid droplet formation in mammary epithelial cells, thus maybe allowing for increased production of milk fat through enhanced lipogenic activity in mammary epithelial cells [41]. This bioactive lipid’s regulatory effect on lipid synthesis underscores its important role in milk fat production.

In contrast, certain plasma metabolites (2-amino-1,3,4-octadecanetriol, 3-(2-hydroxyethyl)indole, and 3-hydroxy-L-proline) were related to reducing milk fat content. 2-Amino-1,3,4-octadecanetriol, a sphingoid base, is thought to interfere with lipid synthesis by disrupting normal lipid metabolic pathways. Sphingoid bases can inhibit the accumulation of lipids in mammary cells, likely resulting in reduced availability of lipid components essential for milk fat synthesis [42].

MA supplementation in lactating cows affected the plasma biochemical index. Wang et al. have evaluated the feeding effect of MA in Holstein cows during early lactation. They found that the concentrations of NEFA and BHB in plasma were lower for MA-supplemented cows and linearly decreased with increasing MA supplementation [43]. Considering the effect of NEFA and BHB in plasma on milk fat synthesis, MA supplementation may decrease milk fat synthesis.

### 4.3. Effect of Milk Metabolite on Milk Fat Content

Differential metabolites identified by non-targeted metabolomics in milk from cows with different milk fat contents on the same feeding pattern were primarily organic heterocyclic compounds, lipids, and organic acids [44]. This proves the accuracy and reliability of screening the differential metabolites in this study using the random forest method. Steroids and the steroid derivative (Beta-muricholic acid) and hydroxy acid and its derivative (MA) were different metabolites between the L-MF and H-MF groups. β-muricholic acid is a major primary bile acid, biosynthesized by rat and mouse liver, and less common in other animals. Fujita et al. have demonstrated that a decrease in β-muricholic acid suppresses lipid content in mouse liver through an antagonistic effect to the farnesoid X receptor, which interferes with SREBP1c-mediated lipogenesis [45]. Bile acids play an important role in regulating lipid absorption, transport, and metabolism. Under diet-induced milk fat depression in goats, compared with control goats, the content of primary bile acid was significantly decreased in the mammary artery and vein plasma [46]. Therefore, the reduction in primary bile acid concentration in the milk of the L-MF group was likely to be a factor that interferes with milk fat synthesis.

L-carnitine is involved in mitochondrial β-oxidation, and it is also of significance as an acetyl buffer. The acetyl group of acetyl-CoA, which is abundantly produced from lipolysis and breakdown of FAs, is increasingly transferred to L-carnitine, as required [47]. Dietary L-carnitine increased the milk fat content in dairy cows with its effect of stimulating hepatic palmitate β-oxidation, which was accompanied by higher plasma BHB in L-carnitine-treated cows [47]. Servillo et al. have compared carnitine precursors and short-chain acylcarnitines between Italian Mediterranean buffalo milk (high milk fat content) and Holstein cow milk (low milk fat content). They found that buffalo milk contained higher levels of L-carnitine and acylcarnitines than Holstein cow milk [48]. These reports agree with our finding of greater plasma BHB and milk L-carnitine levels in the H-MF group cows.

### 4.4. Metabolite Trend During Rumen Fluid–Plasma–Milk

The variation pattern of metabolites during rumen fluid–plasma–milk from dairy cows with different milk fat contents remains unclear. All of the 211 metabolites in our current analyses could be mainly classified into 8 profiles. The largest numbers of metabolites were classified into profile 1 in the POS and NEG modes, which were down-regulated from the rumen fluid to plasma. Metabolites in the rumen fluid are mainly transferred into the body through the rumen epithelium to play their functions [49]. This may be responsible for the decreased levels of profile 1 metabolites in plasma compared with rumen fluid. And the KEGG enrichment for the metabolites in profile 1 was mainly related to lipid metabolism, so interactions between the rumen microbiome and host metabolism are crucial for milk fat synthesis. 9-oxo-octadecanoic acid specifically enriched in profile 1 of the H-MF group is related to the linoleic acid metabolism. Milk metabolome of dairy cows fed different carbohydrate types from silages and concentrates showed that 9-oxo-octadecanoic acid in milk was one of the most promising predictive compounds of corn silage diets, and its level was down-regulated compared with grass-clover silage diet [50]. Kynurenic acid specifically enriched in profile 1 of the L-MF group is related to the tryptophan metabolism. Yang et al. have studied the rumen and plasma metabolomics profiling associated with increasing dietary corn proportions in the diet of beef steers, and they found that phenylalanine, tyrosine and tryptophan biosynthesis and phenylalanine metabolism were common key metabolic pathways for the two biofluids [51]. Because a high-corn diet could induce milk fat depression, 9-oxo-octadecanoic acid and kynurenic acid may relate to milk fat synthesis of dairy cows. Furthermore, in some animal studies, kynurenic acid has been considered as a significant protector against such metabolic diseases as obesity and nonalcoholic fatty liver disease [52].

The levels of metabolites in profile 3 were similar between rumen fluid and plasma but down-regulated from plasma to milk. The trend of profile 4 is completely opposite to that of profile 3, which showed up-regulation from plasma to milk. The KEGG enrichment for the metabolites in profile 3 was mainly related to amino acid metabolism, such as valine, leucine and isoleucine biosynthesis, and biosynthesis of amino acids. Meanwhile, the pathways associated with profile 4 appeared to be associated mainly with energy metabolism, such as carbohydrate digestion and absorption, as well as starch and sucrose metabolism. It was reported that blood metabolites play an important role in regulating the milk yield and milk quality of dairy cows. Blood metabolites could influence the energy and amino acid metabolism during the milk production process in Holstein cows [53], which is consistent with our finding.

In the mammary glands of dairy cows, amino acid uptake from blood is almost equal to milk output based on a nitrogen basis. However, some amino acid uptake:output ratios could be larger than 1 (valine, isoleucine, leucine and arginine), which could be metabolized to CO_2_, urea, polyamine or simply other non-essential amino acids [54]. The flow of essential amino acids at different sites in dairy cows showed that 6 amino acids, including histidine, isoleucine, leucine, methionine, phenylalanine, and lysine, were decreased from portal absorption to milk [55]. These downward trends are consistent with our finding, which shows that the pathway of valine, leucine and isoleucine biosynthesis was down-regulated from plasma to milk. In addition, milk component synthesis is a physiological process with a high energy requirement [53]. On the mammary gland nutrient supply level, energy has been thought of being capable of increasing milk protein production, so the trends of some amino acid metabolism and energy metabolism were reversed from plasma to milk [55].

Several studies have demonstrated that some functional amino acids not only participate in milk protein synthesis but also regulate milk fat synthesis [54,56]. Methionine, lysine, and leucine stimulate the expression, phosphorylation, and nuclear localization of glycyl-tRNA synthetase for transcription of related lipogenic genes [56]. In the H-MF group, L-tyrosine and L-phenylalanine, involved in aminoacyl-tRNA biosynthesis, phenylalanine metabolism, and protein digestion and absorption, showed higher levels in the vein plasma than in the milk. Higher concentrations of tyrosine and phenylalanine were detected in the artery compared to the vein of dairy cows, and the biosynthesis of these amino acids is not found in mammary glands [57]. Taken together, the mammary glands of the H-MF cows may take up less L-tyrosine and L-phenylalanine than the L-MF cows. Phenylalanine was first identified as a potentially limiting amino acid in lactating dairy cows, and supplementing phenylalanine to dairy cows increased body condition scores through reducing fat mobilization [58]. Many studies have mentioned that reduced fat mobilization results in lower availability of substrates for mammary fat synthesis [59,60]. In agreement with this, phenylalanine may affect milk fat synthesis, but the regulation mechanism of phenylalanine needs to be implemented in the future.

Regarding profile 4, MA was specifically found in the L-MF group, and it was also a differential metabolite in milk between the L-MF and H-MF groups. The concentration of MA in the milk of the L-MF group was higher than the H-MF group, but the concentrations of MA in the rumen fluid or plasma did not differ between the two groups, which means that the mammary epithelial cells in the L-MF group might absorb more MA from plasma or synthesize more MA in the mammary gland tissue.

### 4.5. MA Reduced Lipogenesis in BMECs

In our study, a 200 μM MA supplementation in BMECs decreased TAG content by altering the mRNA abundance of genes encoding lipogenic enzymes. The coordinated nature of the deregulation, which tended to affect most of the metabolic pathways studied, including fatty acid synthesis (*FASN*), fatty acid desaturase (*SCD*), lipogenesis (*ME1*) and the transcriptional regulation factor (*SREBP1*), supported the involvement of MA as an important regulator of milk fat synthesis.

FASN is involved in de novo synthesis of medium-chain fatty acids (MCFAs) in mammary glands, providing a substrate for TAG synthesis. A reduction in the expression level of *FASN* decreased the synthesis of C10:0 and C12:0 in goat mammary glands [61]. Acetyl-CoA is the substrate of FASN, and the two-carbon units from malonyl-CoA are added to the fatty acyl chain to generate longer chains. Each cycle requires two molecules of NADPH (Figure 7D). So *FASN* needs NADPH, with acetyl-CoA and malonyl-CoA as precursors, to catalyze the de novo synthesis of FAs [62].

ME1 supports lipogenesis, cholesterol synthesis, and cellular redox potential by catalyzing the decarboxylation of L-Malic acid (L-MA) to pyruvate and the concomitant reduction of NADP to NADPH. Furthermore, ME1 can catalyze reversible reactions [63,64]. Lower mRNA expression levels of *ME1* indicated the lower level of NADPH, so this may relate with the reduction in the *FASN* expression level in MA-treated BMECs in this study. However, the underlying mechanism of MA that decreased the mRNA expression level of *ME1* remains unclear. The most likely explanation for the inhibitory effect of MA on *ME1* was the difference in the structure of substrate: DL-MA vs. L-MA. DL-MA was used in this study.

*SREBP1* is synthesized as an inactive precursor in the endoplasmic reticulum and forms a complex with the SREBF partner (SCAP), which activates the lipogenic genes after being hydrolyzed in the Golgi apparatus and indirectly regulates milk fat synthesis [65]. *SREBP1* is a key transcription factor governing TAG synthesis in ruminants, primarily by activating genes involved in FA and TAG synthesis, such as *SCD*, acetyl-CoA carboxylase (*ACC*), and *FASN* [66]. Xu et al. have used siRNA technology to knock down *SREBP1* in goat mammary epithelial cells and observed that the concentration of TAG was significantly decreased, along with the mRNA expression level of *SCD* and *FABP3* [67]. The down-regulation of de novo synthesis of FAs (FASN) and the transcriptional regulation factor (SREBP1) support the involvement of MA as a central regulator of milk fat synthesis (Figure 7D).

The activity of PPARγ could represent an important control point of milk TAG synthesis in bovine mammary and adipose cells [67]. It has been reported that *PPARG* significantly promoted lipid synthesis in goat mammary epithelial cells, and its mRNA expression can be activated by LCFA [68,69]. In our study, MA promoted the expression of *PPARγ*, while dramatically decreasing the TAG content in BMECs. Thus, we speculated that MA may alter the FA profile in BMECs, such as decreasing the MCFA content through de novo synthesis, increasing the LCFA content, which offers ligands for binding and activating PPARγ. Such an effect may be a reason for the increase in the gene and protein expression level of PPARγ after MA supplementation.

## 5. Conclusions

This study revealed that milk fat content in Chinese Holstein cows is intricately linked to metabolic profiles across the rumen fluid, plasma, and milk, reflecting coordinated interactions between ruminal fermentation, systemic nutrient delivery, and mammary gland synthesis. Compared to L-MF cows, H-MF cows exhibited elevated ruminal FAs (e.g., lauric acid) and plasma BHB, alongside increased milk L-carnitine, suggesting enhanced lipogenic substrate availability to the mammary glands and the FA β-oxidation capacity in the whole body. In contrast, L-MF cows showed higher levels of ruminal citraconic and orotic acids, plasma 3-hydroxy-L-proline, and milk MA, indicative of metabolic stress, tissue catabolism, and altered lipid metabolism. The in vitro experiments demonstrated that 200 μM of MA reduced TAG synthesis in BMECs by down-regulating key lipogenic genes (*FASN*, *SCD*, *SREBP1*, and *ME1*). These findings underscore the critical role of multi-tissue metabolic interactions in regulating milk fat synthesis and highlight MA as a pivotal modulator of mammary lipid metabolism, offering potential insights into optimizing dairy cow nutrition and milk quality.

## Figures and Tables

**Table 1:** Gradient elution condition used in UHPLC.

Time	Eluent A (%)	Eluent B (%)
0.0	98	2
1.5	98	2
12.0	0	100
14.0	0	100
14.1	98	2
17.0	98	2

The metabolome data analysis procedure was conducted as previously described. Briefly, data were processed by the Compound Discoverer 3.1 (Thermo Fisher, Waltham, MA, USA) software, and compounds were identified based on additive ions, molecular ion peaks, and fragment ions. The qualitative and relative quantitative analysis of metabolites were performed based on mzCloud, mzVault, and MassList databases.

**Table 2:** Comparative analysis of plasma biochemical index of dairy cows with different milk fat contents.

Item	Group		*p*-Value
	L-MF	H-MF	
Glucose (mmol/L)	3.56 ± 0.06	3.44 ± 0.22	0.61
TAG (mmol/L)	0.18 ± 0.07	0.12 ± 0.01	0.41
Total cholesterol (mmol/L)	4.39 ± 0.23	4.65 ± 0.54	0.67
High-density lipoprotein cholesterol (mmol/L)	2.22 ± 0.07	2.25 ± 0.04	0.76
Low-density lipoprotein cholesterol (mmol/L)	1.76 ± 0.13	1.95 ± 0.40	0.67
BHB (μmol/L)	473.32 ± 4.10	495.66 ± 6.77	0.02
NEFA (mmol/L)	1.78 ± 0.20	1.92 ± 0.20	0.63

**Table 3:** Random forest classification results of metabolomics data.

Sample	Models	Predictive Accuracy (%)	Area Under ROC Curve
Rumen fluid	POS	40.00	0.40
	NEG	80.00	0.96
Plasma	POS	70.00	0.60
	NEG	60.00	0.84
Milk	POS	70.00	0.76
	NEG	60.00	0.76

**Table 4:** Differential metabolites in rumen fluid, plasma and milk from dairy cows with different milk fat contents (MDA > 0.004 and *p* < 0.05).

Sample	Metabolites	Formula	Retention Time (min)	*m*/*z*	log_2_ FC	*p*-Value
Rumen fluid	Octadecanedioic acid	C_18_H_34_O_4_	14.49	315.25	0.57	0.03
	Lauric acid	C_12_H_24_O_2_	13.82	199.17	0.59	<0.01
	Succinic acid	C_4_H_6_O_4_	1.23	135.03	0.73	0.01
	Citraconic acid	C_5_H_6_O_4_	1.23	129.02	−0.38	<0.01
	3-Hydroxybutyric acid	C_4_H_8_O_3_	1.46	103.04	−0.30	0.02
	Lauric acid ethyl ester	C_14_H_28_O_2_	14.49	227.20	0.95	0.01
	12(13)-DiHOME	C_18_H_34_O_4_	12.59	313.24	0.79	0.03
	N1-[4-(2-thienylthio)phenyl]-4-chlorobenzamide	C_17_H_12_ClNOS_2_	1.16	344.00	−0.39	0.01
	Orotic acid	C_5_H_4_N_2_O_4_	1.45	155.01	−0.44	0.02
Plasma	2-Amino-1,3,4-octadecanetriol	C_18_H_39_NO_3_	13.08	318.30	−0.84	<0.01
	3-(2-Hydroxyethyl) indole	C_10_H_11_NO	9.94	162.09	−0.27	0.01
	Methionine sulfoxide	C_5_H_11_NO_3_S	2.11	166.05	0.41	0.01
	3-Hydroxy-L-proline	C_5_H_9_NO_3_	1.90	132.07	−0.86	0.02
	PC (20:0/20:5)	C_48_H_86_NO_8_P	15.53	894.63	1.28	<0.01
	DL-Arginine	C_6_H_14_N_4_O_2_	1.43	173.10	0.48	0.02
	LPE 18:3	C_23_H_42_NO_7_P	14.38	474.26	0.56	0.03
Milk	L(-)-Carnitine	C_7_H_15_NO_3_	1.35	162.11	0.47	0.02
	Beta-Muricholic acid	C_24_H_40_O_5_	13.57	409.29	0.85	0.02
	DL-Malic acid	C_4_H_6_O_5_	1.21	133.01	−0.59	0.02
	Trehalose 6-phosphate	C_12_H_23_O_14_P	1.28	421.07	0.84	0.03

**Table 5:** Differential metabolites between L-MF and H-MF groups belong to significantly enriched profiles in the trend analysis.

Profile	Group	Metabolites	Class	KEGG
POS Profile 1	L-MF	8-Hydroxyquinoline	--	--
		2-Amino-1,3,4-octadecanetriol	--	--
		Kynurenic acid	Quinolines and derivatives	Tryptophan metabolism
		19-Nortestosterone	Steroids and steroid derivatives	--
		DL-Stachydrine	--	--
		Diphenylamine	Benzene and substituted derivatives	--
		4-oxo-4-(5,6,7,8-tetrahydronaphthalen-1-ylamino)but-2-enoic acid	--	--
	H-MF	Nicotinic acid	Carboxylic acids and derivatives	Pertussis
		5,6-dimethoxy-2-(2-methoxyphenyl)-4H-chromen-4-one	--	--
		9-oxo-octadecanoic acid	Fatty acyls	Linoleic acid metabolism
		5-acetyl-2,6-dimethyl-1,2,3,4-tetrahydropyridin-4-one	--	--
		4-Nitrosodiphenylamine	--	--
POS Profile 3	L-MF	LPC 18:3	--	--
	H-MF	2-Hydroxycinnamic acid	Cinnamic acids and derivatives	Phenylalanine metabolism, biosynthesis of secondary metabolites
		L-Tyrosine	Carboxylic acids and derivatives	aminoacyl-tRNA biosynthesis, phenylalanine metabolism, protein digestion and absorption
		L-Phenylalanine	Carboxylic acids and derivatives	aminoacyl-tRNA biosynthesis, phenylalanine metabolism, and protein digestion and absorption
		D-(+)-Proline	--	--
NEG Profile 4	L-MF	DL-Malic acid	Hydroxy acids and derivatives	--
		Methylsuccinic acid	Fatty acyls	--
		D-(+)-Maltose	Organooxygen compounds	Carbohydrate digestion and absorption, starch and sucrose metabolism, and ABC transporters
	H-MF	Ethyl-D-glucuronide	--	--

## Data Availability

All data generated and analyzed during this study are included in this published article. Raw data supporting the findings of this study are available from the corresponding author on request.

## Supplementary Materials

The following supporting information can be downloaded at: https://www.mdpi.com/article/10.3390/ani16081136/s1, Table S1: Ingredient and nutrient composition of the diet. Table S2: Milk fat content of the selected cows in the dairy farm from August 2020 to January 2021. Table S3: Primers for RT-qPCR. Table S4: Differential metabolites in rumen fluid from dairy cows with different milk fat contents (fold change ≠ 1 and *p* < 0.05). Table S5: Differential metabolites in plasma from dairy cows with different milk fat contents (fold change ≠ 1 and *p* < 0.05). Table S6: Differential metabolites in milk from dairy cows with different milk fat contents (fold change ≠ 1 and *p* < 0.05). Table S7: Metabolites enriched in the trend analysis (POS mode). Table S8: Metabolites enriched in the trend analysis (NEG mode). Figure S1: Milk fat content of lactating cows in the dairy farm from August 2020 to January 2021. Autumn, August to October; winter, November to January; Avg, average milk fat content. Red spot represents H-MF, and blue spot represents L-MF. L-MF, low milk fat content group; H-MF, high milk fat content group. Figure S2: Distribution of Cook’s distance for milk fat content of all dairy cows in autumn and winter from August 2020 to January 2021. A, B respectively represent autumn and winter. The dotted line indicates the reference threshold of 4/*n* = 0.4. Figure S3: Venn diagram illustrating overlap of metabolites enriched in trend analysis.
