# Supplementary figures and images for: Rumen–Plasma–Milk Metabolomics Profiling Revealed Metabolic Alterations Associated with Milk Fat Synthesis in Chinese Holstein Cows
Source: Animals (Basel). 2026 Apr 8;16(8):1136. doi: 10.3390/ani16081136 (PMC13114202; doi:10.3390/ani16081136)

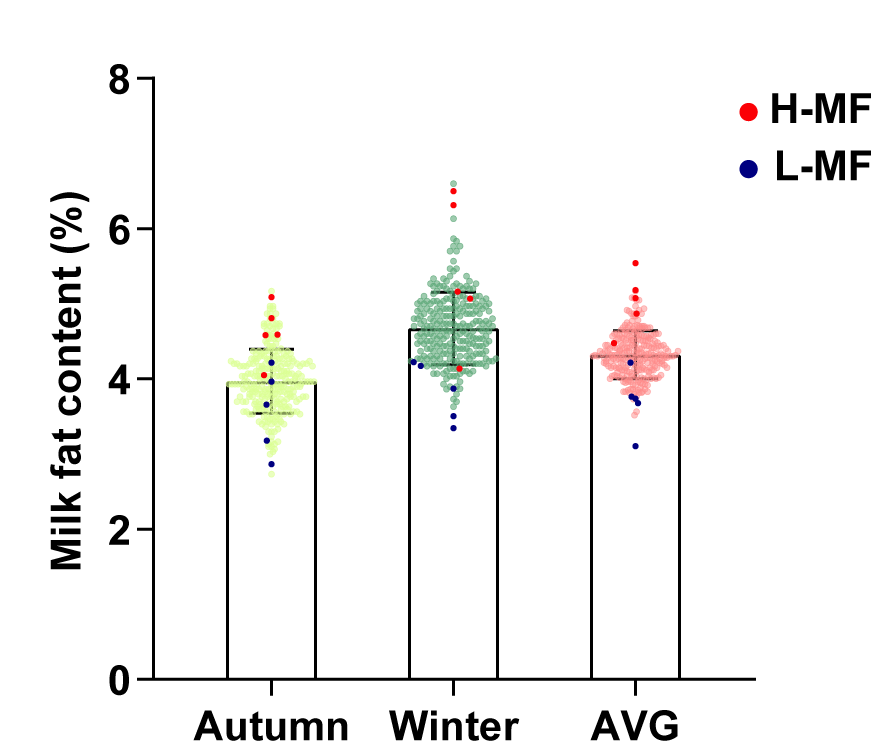

Supplement: Supplementary file 1 [file animals-16-01136-s001.zip › Figure S1.tif]

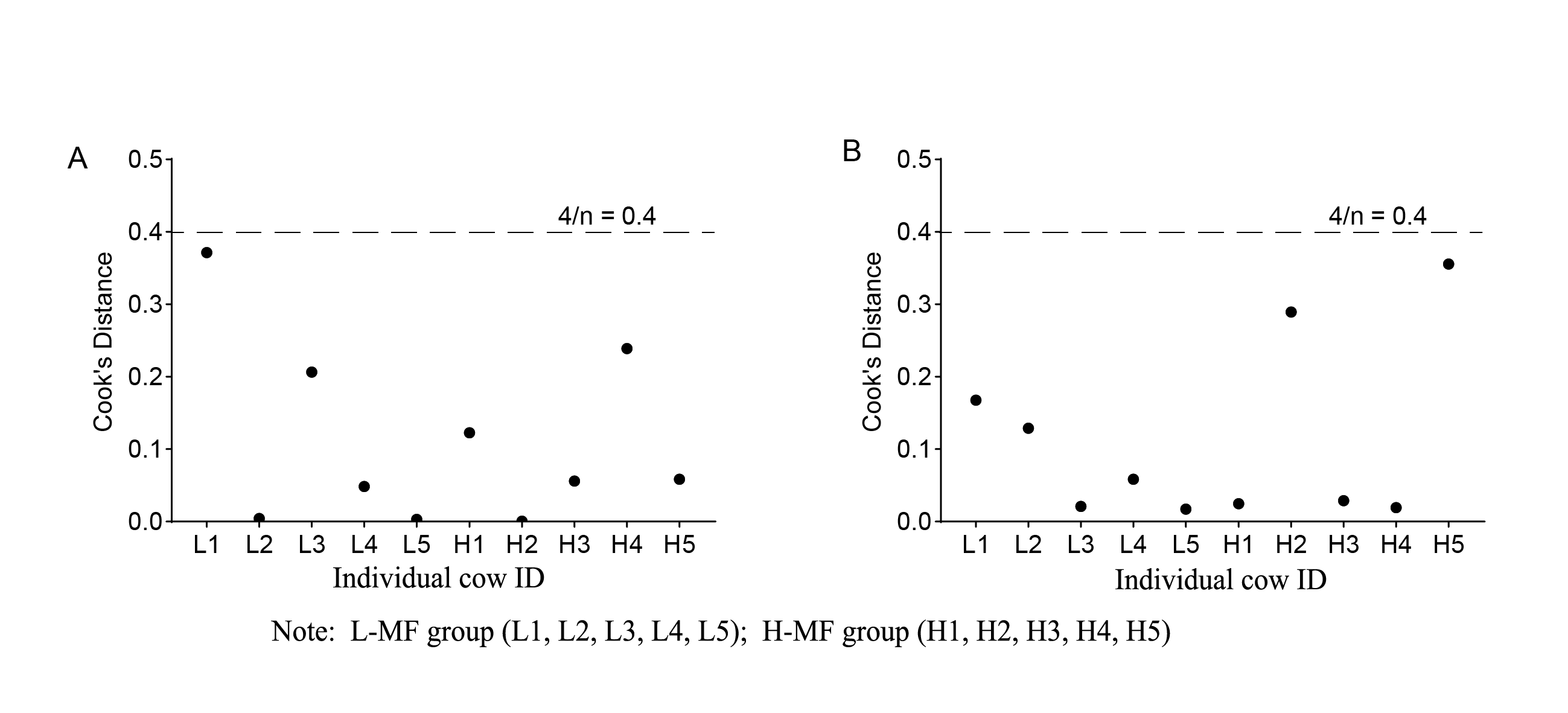

Supplement: Supplementary file 1 [file animals-16-01136-s001.zip › Figure S2.tif]

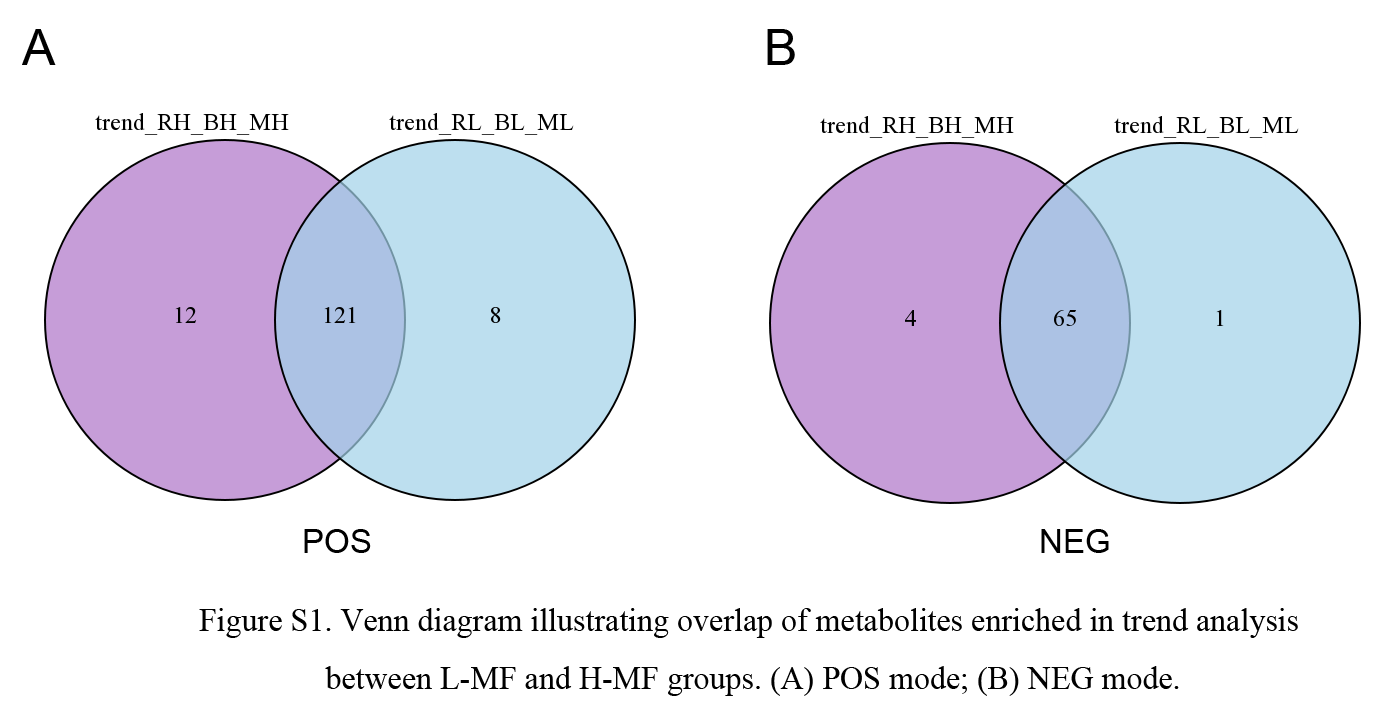

Supplement: Supplementary file 1 [file animals-16-01136-s001.zip › Figure S3.tif]
